# Hepatogenous Photosensitivity Outbreak after Coccidiosis in Grazing Holstein Steers

**DOI:** 10.3390/vetsci7040186

**Published:** 2020-11-24

**Authors:** Christine C. Nieman, Daniel M. Schaefer, Michael Maroney, Kathryn Nelson, Kenneth A. Albrecht

**Affiliations:** 1 USDA-ARS Dale Bumpers Small Farms Research Center, AR-23, Booneville, AR 72927, USA; 2 Department of Animal Sciences, University of Wisconsin-Madison, 1675 Observatory Drive, Madison, WI 53706, USA; schaeferd@ansci.wisc.edu; 3 Research Animal Resources and Compliance, University of Wisconsin-Madison, 1710 University Avenue, Madison, WI 53726, USA; maroney@rarc.wisc.edu (M.M.); knelson@rarc.wisc.edu (K.N.); 4 Department of Agronomy, University of Wisconsin-Madison, 1575 Linden Drive, Madison, WI 53706, USA; kaalbrec@wisc.edu

**Keywords:** photosensitivity, coccidiosis, hepatic lipidosis, grazing, holstein, pasture, phylloerythrin, photodynamic

## Abstract

Clinical signs of photosensitivity in cattle can occur sporadically and unpredictably. It is believed that cases of photosensitivity may be underreported, causing inaccurate and inflated reports of mortality. Additionally, because secondary photosensitization in grazing cattle occurs with liver damage or dysfunction, photosensitivity can have many potential or associated causes. This case links a previous occurrence of coccidiosis to an outbreak of photosensitivity in grazing Holstein steers. Grazing management staff first observed clinical signs of photosensitivity 17 days after an outbreak of coccidiosis and subsequent turnout to spring pastures. Clinical signs were observed in 25% of the population. The severity of photosensitivity was variable and ranged from blistered skin on the muzzle to sloughing of unpigmented epidermis and thinly haired regions. Severely affected cattle were removed from pasture, housed under shade, monitored for infection, and recovered without treatment. Mild cases remained on pasture and recovered without treatment. Photosensitivity did not reoccur in the cattle that remained on pasture or in mildly affected cattle returned to pasture. Photosensitivity did not appear to be associated with pasture weeds, a specific forage species, or variable or extreme weather conditions that could have resulted in mycotoxin production. The occurrence appears to have been a result of a previous and concurrent coccidiosis outbreak that caused secondary photosensitization through hepatic lipidosis caused by anorexia and dehydration associated with the severe coccidiosis. Although clinical signs appeared suddenly, cattle recovered quickly and without treatment.

## 1. Introduction

Photosensitivity in cattle causes abnormal lesions, blistering, and peeling of skin, which occur on unpigmented epidermis and thinly haired regions [1]. The severity of photosensitivity can range from mild erythema and oedema to fissuring of the epithelium, exudation, crusting of serum, and necrosis and sloughing of skin [1]. Clinical signs have been seen within 24 h of induced cases [1]. On farm, photosensitivity clinical signs have been reported to be observed within 4 days [2] to several weeks [3] after a change in feed source.

There are three types of photosensitization based on etiology: primary or Type 1, direct consumption of photodynamic agent; congenital or Type 2; and hepatogenous or secondary/Type 3 [4]. In Type 1 photosensitization, liver function is normal, and the photodynamic compound is absorbed and present in circulating blood, where a reaction with sunlight occurs [5]. Type 2 is rare and is caused by abnormal heme synthesis, resulting in accumulation of photodynamic metabolites [6]. Hepatogenous photosensitivity is the most common form [7], caused by the accumulation of phytoporphyrin (phylloerythrin) in dermal tissues [8]. Phylloerythrins are the metabolites of chlorophyll produced by microbial fermentation in ruminants [9] and are absorbed by the rumen, abomasum, and intestine, and are normally excreted in the bile [10]. When hepatocellular or biliary damage or biliary stasis is present, excretion of phylloerythrin is impaired and it accumulates to phytotoxic levels [1,8].

Mortality and morbidity due to outbreaks of photosensitization are highly variable in the literature. Photosensitivity is known to occur in grazing cattle throughout the world, is sporadic, and is believed to be low occurrence, though that could be skewed by low reporting [7] and, likely, mild cases go unnoticed. Therefore, the purpose of this report is to share observations of an outbreak of photosensitivity that appeared suddenly, but also resolved quickly.

## 2. Case Description

**Figure 1:**
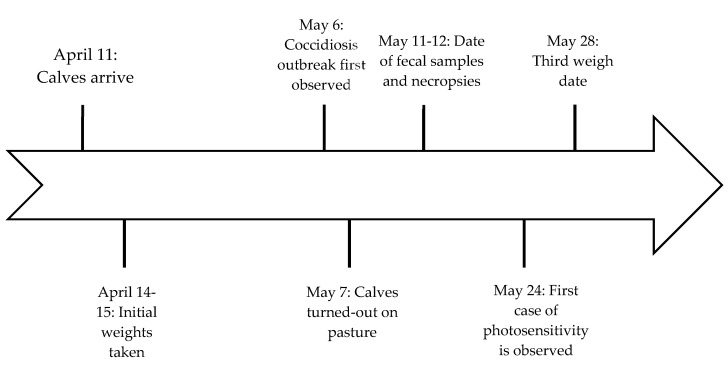
Timeline for important dates related to symptom observation and sampling.

The reported case study occurred unexpectedly during a research project approved by the College of Agricultural and Life Sciences Animal Care and Use Committee (Research Protocol A005360) at the University of Wisconsin-Madison. Cattle were cared for in accordance with the standard operating procedure of the Arlington Beef Center and veterinarians from Research Animal Resources and Compliance oversaw the care of the cattle described in this case report. A timeline for the case description is provided (Figure 1).

On 11 April 2015, 250 steers weighing 225–250 kg arrived at the University of Wisconsin Arlington Agricultural Research Station, near Arlington, WI USA. The cattle supplier accumulated the desired steers for use in a grazing project by the University. The number of farms from which cattle were accumulated and length of time cattle were co-mingled prior to arrival are unknown. The diet of steers prior to arrival was unknown. The 250 steers were placed in five different pens with free access to dry hay and water. No provision was made to provide supplemental grain or coccidiostat. On 14 April, 2015 steers were processed through the chute. The processing protocol included vaccinations against IBR, PI3, BRSV, BVD types 1 and 2 (Bovi-Shield Gold 5, Zoetis Animal Health), clostridial diseases and infectious bovine keratoconjunctivitis (Vision-20/20, Merck Animal Health). Calves were tested for BVD (all negative), rectal temperatures were determined, and lung auscultation was conducted. Fecal samples were taken from 20% of the population and later examined for determination of fecal egg counts. These samples were unremarkable, with some coccidia present, but low counts, and very few strongyle or nematode eggs present.

On 6 May, cattle were reweighed for turnout onto the grazing study. While weighing, it was observed that cattle displayed clinical signs of coccidiosis. Symptoms were watery diarrhea, lack of rumen fill, anorexia, and, in severe cases, bloody diarrhea and dehydration. Calves were weighed and determined to have lost 31.6 kg since 15 April. Severely affected steers (39 steers) were treated with sulfadimethoxine 40% (Albon, Zoetis; 55 mg/kg) and sulfamethazine bolus (Sustain III Bolus; Bimeda; 32.1 g/90 kg BW), and, in addition, tulathromycin (Draxxin, Zoetis; (2.5 mg/kg (1.1 mL/45 kg BW)) was administered if the animal was also determined to have pneumonia diagnosed via auscultation. Severely affected steers remained in pens and consumed hay and supplement and were turned out to pasture later (after 28 May), as their condition improved. The remaining cattle were turned out onto pasture on 7 May. The pasture water system was fitted with an injection system that allowed for the treatment of water for 5 days with amprolium (Corrid, Huvepharma; 10 mg/kg of BW daily). The decision to turnout moderately and mildly affected cattle to pasture was made to remove cattle from contaminated facilities and allow access to clean pastures where cattle were rotated to fresh pastures every 2 days. Pastured cattle were checked at least twice per day to identify individuals that may have worsening symptoms. By 20 May, there were nine deaths and euthanizations of cattle due to coccidiosis—7 from calves in the severely affected group, and 2 calves on pasture. The diagnosis was confirmed through nine of ten fecal samples taken on 12 May, and via fecal samples from three necropsied steers at the University of Wisconsin Veterinary Diagnostics Laboratory. Necropsied steers died on 11 May (1) and 12 May (2). By 20 May, coccidiosis appeared to have passed and cattle were in recovery.

**Figure 2:**
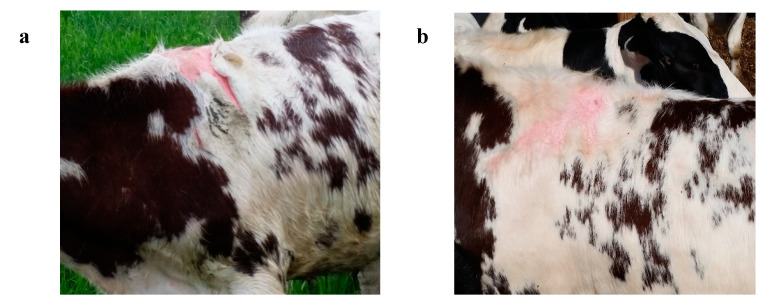
(**a**) Steer with clinical signs of photosensitivity on unpigmented skin. (**b**) Recovered steer from Figure 2a. Hair did not regrow on areas where skin was sloughed.

**Figure 3:**
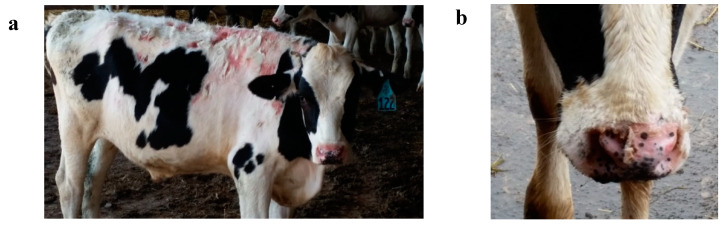
(**a**) Steer with clinical signs of photosensitivity on unpigmented skin. (**b**) Blistering on muzzle caused by photosensitivity.

The first case of photosensitivity was seen on 24 May (Figure 2), 17 days after turnout onto pasture. After discovering several additional cases of photosensitivity, on 28 May, all cattle were weighed and examined for signs of photosensitivity. It was determined that 59 calves or 24.8% of the population displayed clinical symptoms of photosensitivity (Figure 3). Thirteen calves were removed from pasture and housed under shade in confinement. Cattle in this study grazed very lush pasture, mixtures of rye (*Secale cereal* L.)-Kura clover (*Trifolium abiguum* M. Bieb.), alfalfa (*Medicago sativa* L.) -meadow fescue [*Schedonorus pratensis* (Huds.) P. Beauv], alfalfa-tall fescue [*Schedonorus arundinaceus* (Schreb.) Dumort], and alfalfa-orchardgrass (*Dactylis glomerata* L.). Clinical signs of photosensitization were observed on all pasture species. The same study was conducted in the previous year with Holstein steers, and only two cases of photosensitivity were observed. In 2016, the study was repeated and seven cases of photosensitivity were observed. The cause of these cases was not examined and was presumed to be hepatogenous and isolated to these individual calves.

Three necropsies were completed on calves that died from coccidiosis prior to the onset of photosensitivity. The necropsies discovered bile stasis in one steer and hepatic lipidosis in three steers. Three fecal samples from photosensitive steers were also tested for bovine coronavirus, cryptosporidium, bovine rotavirus, and salmonella, and all were negative, except one positive for rotavirus. Phylloerythrin concentrations in blood were not measured.

Cattle with severe cases of photosensitivity were removed from pasture and were not returned to pasture. Calves were monitored for infection, none occurred, and calves recovered without treatment. Moderate cases were removed from pasture until wounds healed, without treatment; these calves were returned to pasture and photosensitivity did not reoccur. Hair did not re-grow in places of photosensitivity (Figure 2b). Mild cases remained on pasture, recovered without treatment, and photosensitivity did not reoccur.

## 3. Discussion

Photosensitivity was believed to be hepatogenous, or Type 3, photosensitivity. Schlegal et al. [11] observed photosensitivity in Holstein steers grazing alfalfa, and determined the cause to be hepatogenous. Other cases of photosensitivity in cattle consuming alfalfa were also determined to be hepatogenous, but involved moldy hay [12] or hay previously exposed to flooding [1,13]. Photosensitivity in this case was observed for all species of pasture including rye-Kura clover mixtures and binary mixtures of grasses and alfalfa. Therefore, pasture species is not believed to be the cause of photosensitivity. However, all species were rapidly growing and would have provided the photodynamic agent. Coccidiosis caused dehydration, diarrhea, and anorexia, requiring the mobilization of adipose tissue and resulting in excessive hepatic lipidosis and liver damage. As seen in early lactation cows in a negative energy balance, fatty acids are mobilized from the adipose tissue to the liver for export, but because the bovine liver has poor capacity to export excess lipids, lipids build up in the hepatocytes [14]. The relationships between hepatic lipidosis and liver damage are variable [14], but in this case, hepatic lipidosis likely resulted in phylloerythrin entering circulation. Additionally, one necropsy, prior to clinical signs of photosensitivity, described bile stasis in the liver. In healthy cattle, phylloerythrin is excreted in the bile and generally never enters circulation, but when the liver is damaged or bile stasis is present, phylloerythrin enters circulation and causes photosensitivity in unpigmented skin [1].

Although we propose that extreme weight loss caused by the coccidiosis led to hepatic lipidosis, it does not appear that the severity of weight loss was related to the development of photosensitivity. The 59 steers that developed photosensitivity lost 20.4 kg (standard deviation (SD) +/− 15.3), compared to 21.2 kg (SD +/− 19.9) lost by steers that did not develop photosensitivity (weights from 14 April and 7 May). From the dates of 7 May to 28 May, photosensitive steers gained less weight, 17.7 kg (SD +/− 13.3), compared to contemporaries without photosensitivity, which gained 23.8 kg (SD +/− 12.4). However, this difference in weight gain may be more related to differences between animals and their ability to recover from the coccidiosis event, rather than reduction in gain due to photosensitivity.

In many cases, calves with severe coccidiosis were also treated for pneumonia. A total of 41 calves were treated with tulathromycin between the 14 April and 11 May, 2 calves died or were euthanized, 1 calf was re-treated, 5 calves were housed in confinement, and only 4 calves developed photosensitivity. Although, pneumonia could have increased weight loss and slowed recovery in some calves, it does not appear that toxemia or hypoxia associated with pneumonia were factors in the development of photosensitivity. Additionally, we have considered that calves with coccidiosis were treated with sulfadimethoxine. Out of 47 calves treated between the dates of 6 May and 20 May with sulfadimethoxine, only 9 developed photosensitivity, though 11 died or were euthanized prior to the first case of photosensitivity. Only 9 of the 59 calves that developed photosensitivity were ever treated with sulfadimethoxine or tulathromycin, and therefore, previous treatments for pneumonia and/or coccidia did not contribute to the development of photosensitivity. Treated calves were housed in confinement for close monitoring and treatment, did not have access to pasture, and did not develop photosensitivity. It is unknown whether those calves in confinement would have developed symptoms, but it appears that there is no relationship between sulfadimethoxine treatment or pneumonia treatments and photosensitivity in these calves.

Another interesting discussion in this case is the timeline. The first observations of coccidiosis occurred on or around 6 May (25 days after arrival) and calves were turned out to pasture on 7 May. Previous reports on photosensitivity have observed clinical signs by 24 h post-surgery in bile duct ligated cattle fed contaminated hay and 4 days post-surgery in cattle grazing fresh pasture after bile duct ligation [1]. However, photosensitivity was not observed in these calves until 24 May, 17 days after exposure to fresh pasture. Early, clinical signs of photosensitivity are difficult to detect in the field, this likely leads to under-reporting of cases as suggested by Chen et al. [7]. In this study, electric fencing was used to contain cattle; other than a salt-mineral feeder and water tank, calves did not have access to a physical object to scratch off sloughing skin. Therefore, staff did not notice any photosensitivity until skin had deteriorated enough that sloughing occurred without contact.

## 4. Conclusions

Other than removing calves with severe cases from pasture into the shade in confinement, calves were not treated for photosensitivity. On 28 May, other than displaying after effects of photosensitivity, calves appeared to be healthy with normal eating behavior and good body condition. Calves did not have elevated temperatures, photosensitive areas were not sensitive to touch, and newly regenerated skin was developed under the sloughing skin. Additionally, no icterus was observed in any of the affected cattle. Glenn et al. [1] observed icterus in all cattle that developed photosensitivity and did not observe photosensitivity in any animals after the icterus disappeared. Therefore, by the time the grazing management staff noticed clinical signs of photosensitivity, likely, the liver was already healed from the hepatic lipidosis and calves were in recovery. Although the authors do not know of any previous cases or bibliographic references that relate hepatic lipidosis with hepatogenous photosensitivity in grazing steers, the findings of this outbreak indicate that they are potentially related. Regardless, in this case, photosensitivity appeared suddenly and cattle recovered without treatment, other than re-location to shade, and there was no occurrence of mortality associated with photosensitivity.

## References

[1] Glenn B.L., Panciera R.J., Monlux A.W. (1965). A hepatogenous photosensitivity disease of cattle: II. Histopathology and pathogenesis of the hepatic lesions. Pathol. Vet..

[2] Witte S.T., Curry S.L. (1993). Hepatogenous photosensitization in cattle fed a grass hay. J. Vet. Diagn. Investig..

[3] Casteel S.W., Weaver A.D., Mills L.L., Pace L.W., Rottinghaus G.E., Smith K.M. (1991). Photosensitization outbreak in shorthorn calves in Missouri. J. Vet. Diagn. Investig..

[4] Quinn J.C., Kessell A., Weston L.A. (2014). Secondary plant products causing photosensitization in grazing herbivores: Their structure, activity and regulation. Int. J. Mol. Sci..

[5] Campbell W.M., Dombroski G.S., Sharma I., Partridge A.C., Collettt M.G. (2010). Photodynamic chlorophyll a metabolites, including phytoporphyrin (phylloerythrin), in the blood of photosensitive livestock: Overview and measurement. N. Z. Vet. J..

[6] Mauldin E.A., Peters-Kennedy J., Maxie M.G. (2016). Integumentary system. Jubb, Kennedy, and Palmer’s Pathology of Domestic Animals.

[7] Chen Y., Quinn J.C., Weston L.A., Loukopoulos P. (2019). The aetiology, prevalence and morbidity of outbreaks of photosensitisation in livestock: A review. PLoS ONE.

[8] Marten G.C., Ehle F.R., Ristau E.A. (1987). Performance and photosensitization of cattle related to forage quality of four legumes 1. Crop Sci..

[9] Rimington C., Quin J.I. (1934). Studies on the photosensitisation of animals in South Africa. 7. The nature of the photosensitising agent in Geeldikkop. Onderstepoort J. Vet. Sci. Anim. Ind..

[10] Rothemund P., McNary R.R., Inman O.L. (1934). Occurrence of decomposition products of chlorophyll. II. Decomposition products of chlorophyll in the stomach walls of herbivorous animals. J. Am. Chem. Soc..

[11] Schlegel M.L., Wachenheim C.J., Benson M.E., Black J.R., Moline W.J., Ritchie H.D., Schwab G.D., Rust S.R. (2000). Grazing methods and stocking rates for direct-seeded alfalfa pastures: I. Plant productivity and animal performance. J. Anim. Sci..

[12] Scruggs D.W., Blue G.K. (1994). Toxic hepatopathy and photosensitization in cattle fed moldy alfalfa hay. J. Am. Vet. Med. Assoc..

[13] Putnam M.R., Qualls C.W., Rice L.E., Dawson L.J., Edwards W.C. (1986). Hepatic enzyme changes in bovine hepatogenous photosensitivity caused by water-damaged alfalfa hay. J. Am. Vet. Med. Assoc..

[14] Cebra C.K., Garry F.B., Getzy D.M., Fettman M.J. (1997). Hepatic lipidosis in anorectic, lactating Holstein cattle: A retrospective study of serum biochemical abnormalities. J. Vet. Intern. Med..

